# Occludin Is Essential to Maintain Normal Alveolar Barrier Integrity and Its Protective Role During ARDS Progression

**DOI:** 10.3390/ijms252111595

**Published:** 2024-10-29

**Authors:** Xin Lin, Haiqing Bai, Michael Barravecchia, Rosemary Norman, Gillian M. Schiralli Lester, R. Matthew Kottmann, Antony Leonard, Arshad Rahman, Jennifer L. Young, David A. Dean

**Affiliations:** 1 Department of Pediatrics, School of Medicine and Dentistry, University of Rochester, 601 Elmwood Avenue BOX 850, Rochester, NY 14642, USA; xinlin.lynn@gmail.com (X.L.); hbai@xellarbio.com (H.B.); michael_barravecchia@urmc.rochester.edu (M.B.); rosemary_norman@urmc.rochester.edu (R.N.); gillian_schirallilester@urmc.rochester.edu (G.M.S.L.); leonard.antony@yahoo.com (A.L.); arshad_rahman@urmc.rochester.edu (A.R.); jennifer_young@urmc.rochester.edu (J.L.Y.); 2 Department of Pathology, School of Medicine and Dentistry, University of Rochester, 601 Elmwood Avenue BOX 850, Rochester, NY 14642, USA; 3 Department of Medicine, School of Medicine and Dentistry, University of Rochester, 601 Elmwood Avenue, Rochester, NY 14642, USA; matt_kottmann@urmc.rochester.edu

**Keywords:** ARDS, acute lung injury, occludin, barrier dysfunction, electroporation, gene therapy

## Abstract

Acute respiratory distress syndrome (ARDS) is a severe lung condition without targeted therapy that is characterized by the disruption of epithelial and endothelial barriers. The role of the tight junction protein occludin in the pathogenesis of this disease is unknown, although it has previously been deemed redundant in some tissues. The aim of the present study is to determine whether occludin is required for lung function by controlling alveolar barrier integrity in mouse models. Immunofluorescence staining of lungs from ARDS patients revealed a significant decrease in occludin expression compared to controls. Gene delivery of shRNA against occludin in the mouse lung reduced occludin levels and induced lung injury, as assessed by wet-to-dry-ratio, histology, and cellularity and protein content of bronchial alveolar lavage fluid. Conversely, gene delivery of an occludin-expressing plasmid increased occludin expression and dampened endotoxin-induced lung injury. In primary rat alveolar epithelial cells, occludin levels were positively correlated with barrier integrity, as well as membrane localization of claudin-18, another tight junction protein. Collectively, our data demonstrate that occludin plays a significant role in alveolar barrier function and that targeting occludin may provide a new therapeutic approach for ARDS.

## 1. Introduction

Acute respiratory distress syndrome (ARDS) is a severe lung disease with up to 45% mortality [1]. It can be caused by a number of underlying conditions, including pneumonia, aspiration of gastric contents, trauma, and sepsis [2]. These factors impair the alveolar epithelial and endothelial barriers, induce inflammation and pulmonary edema, and eventually lead to hypoxia and multi-organ failure. Current treatment for ARDS is limited to supportive care. A better understanding of its pathogenesis is a prerequisite to the development of more effective therapies.

One of the main pathologic features of ARDS is the disruption of the alveolar epithelial and endothelial barriers [3]. Intact function of these barriers is maintained by the tight junction (TJ), the apical-most structure at cell-cell contacts. The TJ is composed of multiple interacting transmembrane and cytoplasmic proteins that are linked to the actin cytoskeleton. The first identified transmembrane protein at the TJ is occludin, a 65 kDa tetra-spanning protein with two short extracellular loops [4]. Decreased expression of occludin has been observed in airway epithelial cells treated with house dust mite [5] and alveolar epithelial cells exposed to hypoxia [6,7], stretch [8], and ventilation [9]. However, whether such a decrease contributes to the disruption of barrier integrity and impairment of lung function is not clear. Moreover, previous studies failed to establish whether occludin is needed for epithelial barrier functioning, especially in the lung. Occludin-deficient embryonic stem cells retain the ability to differentiate into polarized epithelial cells that bear normal TJ [10,11]. In contrast, occludin-knockout mice exhibit impaired junctional formation and increased susceptibility to injuries in the ear, colon, gut, and liver, and to a lesser extent in other organs [12,13]. Knockdown of occludin in Madin–Darby Canine Kidney (MDCK) cells results in an increase in permeability to large organic cations [14]. Hence, occludin seems to show tissue-specific severity in epithelial barrier function. In this study, we aim to investigate the role of occludin in the progression of acute lung injury and explore its utility as a therapeutic target. By using primary alveolar epithelial cells and animal models of ARDS, we demonstrate that occludin is indeed required for normal lung barrier function. Modulating its abundance may lead to new therapies for ARDS. 

## 2. Results

### 2.1. Occludin Expression Is Significantly Decreased in Patients with ARDS

**Figure 1:**
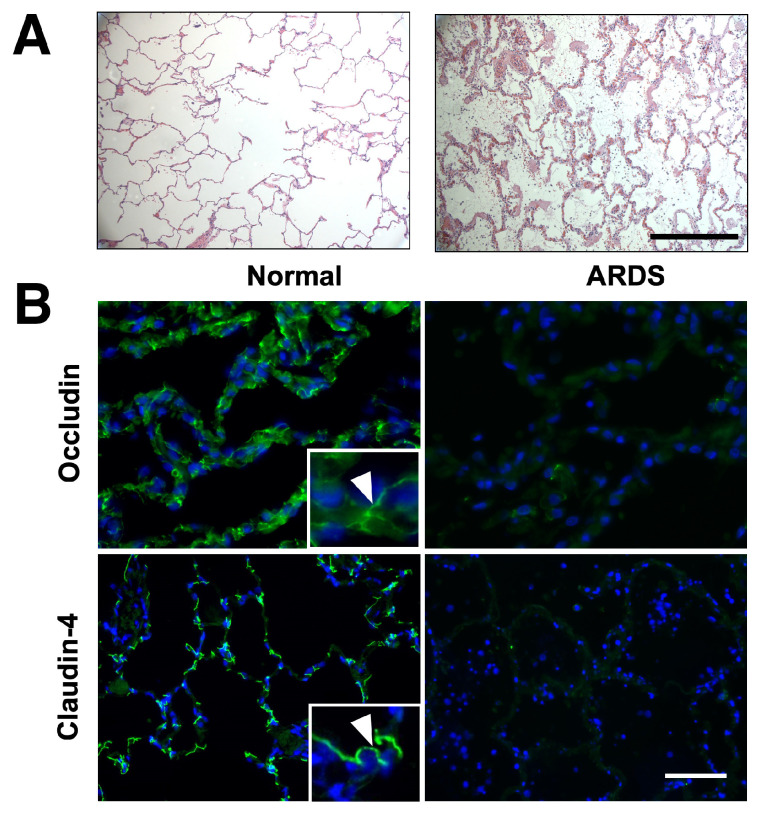
Histology and tight junction protein expression in ARDS lungs. (**A**) Representative H&E staining of lung sections used for immunofluorescence staining. Scale bar: 100 µm. (**B**) The expression of occludin and claudin-4 are decreased in the lungs from patients with ARDS compared with healthy controls. Representative images show reduced occludin and claudin-4 expression in lung sections from ARDS patients by immunofluorescence staining (green). Blue: DAPI staining for the nucleus. Insets are magnified regions of each panel showing membrane localization of the tight junction proteins. Arrows indicate the junctional localization of the staining. Scale bar: 40 µm.

Although previous experimental studies using cells and animals have observed the reduced expression of TJ proteins upon lung injury, such as lipopolysaccharide (LPS) treatment [15,16], no evidence has revealed the alterations of tight junctions by directly analyzing their expression in lungs of ARDS patients. Occludin and claudin-4 are the predominant integral TJ proteins expressed in the alveolar epithelium [17]. Therefore, we used immunofluorescence staining to compare their expression in lung sections from four ARDS patients and four control subjects. Haemotoxylin and Eosin (H&E) staining (Figure 1A) shows major histologic changes in ARDS samples that are not observed in controls, including diffuse alveolar damage, thickening of the alveolar wall, lung edema, and infiltrating cells [18]. Immunostaining of occludin in the control alveoli showed strong staining intensity and linear distribution (Figure 1B and Figure S1), indicating an intact lung epithelial barrier. In contrast, the expression of occludin in ARDS patients is greatly decreased and displays an irregular and spotty pattern of distribution, suggesting a disrupted epithelial barrier. Additionally, this deterioration of tight junction expression in ARDS patients is also observed for claudin-4 (Figure 1B). These results demonstrate that the levels of occludin and other TJ proteins are decreased in patients with ARDS.

### 2.2. Expression of Occludin Is Decreased in Mouse Lungs After Gene Delivery of Occludin shRNA by Electroporation

**Figure 2:**
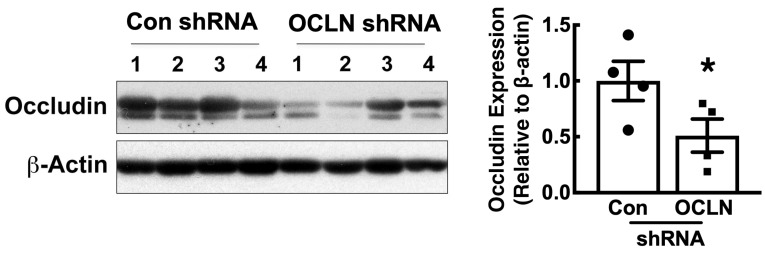
Occludin expression is decreased after electroporation-mediated delivery of occludin shRNA plasmids. Control or occludin shRNA plasmids were delivered to the lungs of mice and, four days later, protein levels were determined in whole lung by Western blot (n = 4). Densitometry was performed and mean ± standard deviation is shown along with the individual animal data points. *, *p* ≤ 0.04 as determined by unpaired *t*-test.

Previous studies have demonstrated that electroporation is a highly effective approach to deliver naked plasmids into live animals for gene overexpression [19,20,21]. When using a Cytomegalovirus (CMV) immediate early promoter to drive overexpression of a transgene in the lung, gene transfer and expression is seen in 33.2% ± 3.5% of parenchymal cells two days after electroporation (18 to 52% in 10 lung sections each from 6 different mice; Figure S2). This gene transfer efficiency is similar to that seen with recombinant Adenovirus, Adeno-associated virus (AAV), or Sendai virus, but unlike the viral vectors, it is not associated with any inflammation. To determine whether the same approach can be used for gene knockdown, we delivered a pool of four shRNA expression plasmids against occludin into mouse lungs. Four days later, expression of occludin was assessed in whole lungs by Western blot. As shown in Figure 2, delivery of the occludin shRNA plasmids by electroporation resulted in a 50–60% reduction of occludin expression in the whole lung lysates. These data indicate that electroporation can be used not only for overexpression of transgenes, but also for knockdown of a gene of interest.

### 2.3. Gene Delivery of Occludin shRNA Can Enhance Lung Injury with or Without LPS Administration

**Figure 3:**
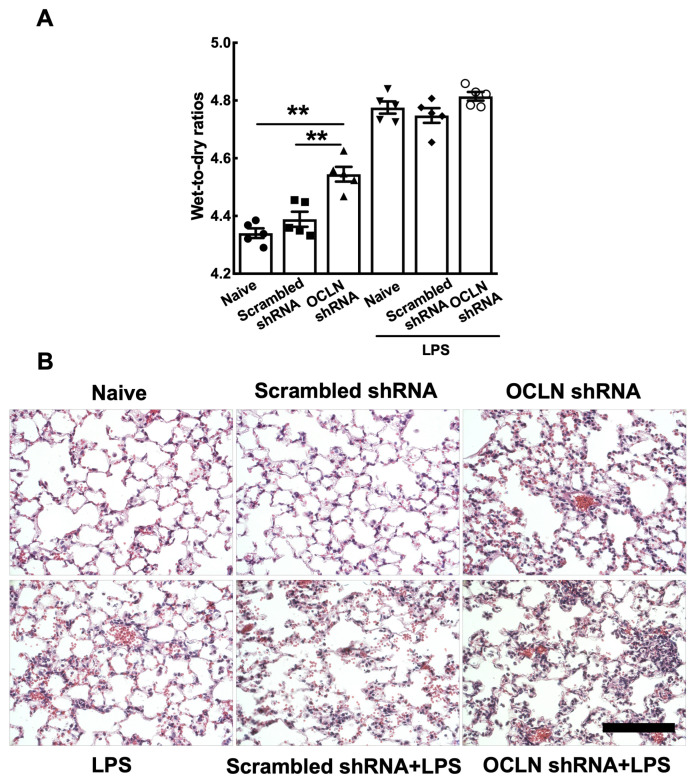
Electroporation-mediated gene delivery of occludin shRNAs can accelerate LPS-induced lung injury. A combination of four distinct occludin shRNA plasmids (160 μg total) in 50 μL was delivered to the lungs by electroporation at 200 V/cm using 8 pulses of 10 msec in duration. Four days after gene delivery, LPS (5 mg/kg) was intratracheally administered to mice, and two days later, lungs were removed for gravimetric analysis. (**A**) Wet-to-dry ratios are shown as mean ± SEM (n = 5). Statistical analysis was by one way ANOVA followed by post-hoc analysis using Tukey’s test, ** *p* < 0.01. (**B**) Hematoxylin and eosin staining were used to compare the histologic features. Scale bar: 100 µm.

To determine the function of occludin in both healthy and injured lungs, occludin shRNA plasmids were electroporated into mouse lungs and, four days later, the lungs were injured by intratracheal administration of LPS (5 mg/kg). After two days, lung injury was evaluated by measurement of wet-to-dry weight ratios, histology, bronchoalveolar lavage (BAL) protein levels, and BAL cellularity. Gene delivery of occludin shRNAs without LPS administration resulted in significant enhancement of lung injury compared with naïve or scrambled shRNA, as measured by wet-to-dry ratios (Figure 3A, 4.55 ± 0.03 vs. 4.34 ± 0.02 or 4.39 ± 0.03, respectively). However, little difference in lung injury was seen after gene delivery of occludin shRNAs with LPS-challenged mice compared to LPS only or LPS-challenged mice that received the control scrambled shRNA. Histologically, evidence of increased edema and inflammation is seen in mice that received occludin shRNA plasmids compared with naïve mice or mice that received the scrambled shRNA, but not in LPS-challenged mice (Figure 3B). 

**Figure 4:**
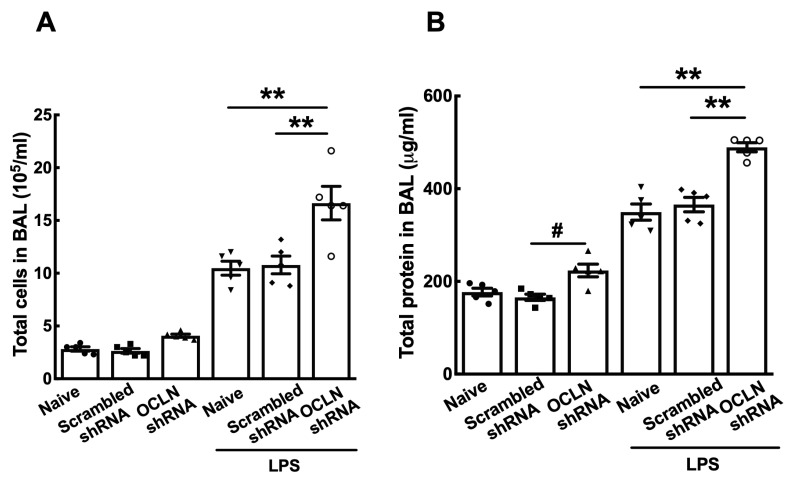
Gene delivery of occludin shRNA plasmids enhances LPS-induced inflammation. A 160 μg combination of 4 distinct occludin shRNA plasmids in 50 μL was delivered to the lungs by electroporation at 200 V/cm using 8 pulses of 10 msec in duration. Four days after gene delivery, LPS (5 mg/kg) was intratracheally administered to mice, and two days later, BAL fluid was collected and assessed (**A**) by cellularity of BAL fluid and (**B**) total protein levels in BAL (n = 5–6). Statistical analysis was by one way ANOVA followed by post-hoc analysis using Tukey’s test. ^#^ *p* < 0.05 and ** *p* < 0.01.

Interestingly, although delivery of occludin shRNAs slightly increased total cell numbers in BAL fluid compared with naïve mice or scrambled shRNA mice, they were not statistically significant (Figure 4A). Contrary to the cellularity of BAL fluid, a significant difference in total protein levels was observed after gene delivery of occludin shRNAs compared with the scrambled shRNA mice (Figure 4B). In contrast, LPS-challenge of mice previously transduced with occludin shRNAs showed increased total numbers of cells in BAL fluid by 58.8% or 54.4% compared to LPS only or LPS-injured mice receiving scrambled shRNA, respectively (Figure 4A). Similarly, total protein levels in BAL fluid from those mice were significantly increased after electroporation of occludin shRNAs (Figure 4B). Taken together, these results demonstrate that reduced expression of occludin in the lung can lead to lung injury and further exacerbate the severity of injury induced by LPS treatment.

### 2.4. Occludin Is Needed for Normal Alveolar Barrier Function In Vitro

**Figure 5:**
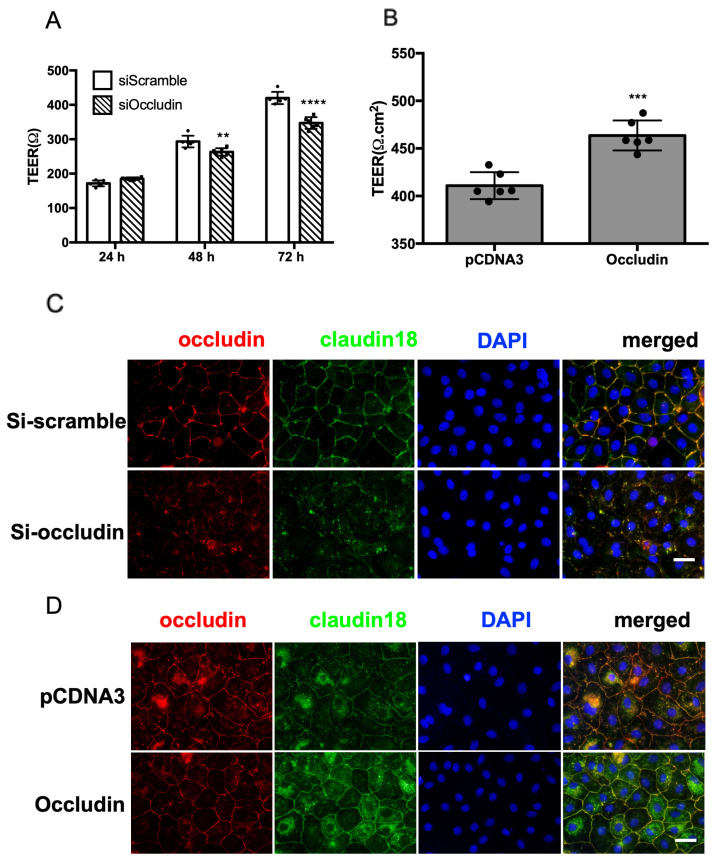
Occludin is needed for alveolar barrier function in vitro. (**A**) Alveolar epithelial type II (ATII) cells transfected with siRNA-occludin or scrambled control were plated in transwell plates. TEER was measured as indicated, post transfection. Statistics were performed using two-way ANOVA with Tukey’s multiple comparisons test. n = 6, ** *p* < 0.01, **** *p* < 0.0001. (**B**) ATII cells transfected with pcDNA3 or occludin plasmid were plated in transwell plates. TEER was measured 48 h after transfection. Statistics were performed using Student’s *t* test (n = 6, *** *p* < 0.001). (**C**) Knockdown of occludin reduces claudin18 localized at tight junctions in ATII cells. ATII cells isolated from rats were electroporated with scramble or occludin shRNA. Then, 72 h later, cells were stained for occludin (red) and claudin18 (green). Nuclei are stained with DAPI (blue). Scale bar: 40 µm. (**D**) Overexpression of occludin induces claudin18 localized at tight junctions in ATII cells. ATII cells isolated from rats were electroporated with empty plasmids or plasmids expressing occludin. Then, 48 h later, cells were stained for occludin (red) and claudin18 (green). Nuclei are stained with DAPI (blue). Scale bar: 40 µm.

Since knockdown of occludin in mice impairs lung function, we next investigated whether this is due to the disruption of alveolar barrier function. Primary rat alveolar type II cells (ATII) were isolated, and 24 h later, transfected with occludin siRNA. Alveolar barrier function was monitored by transepithelial resistance (TEER) measurement at 24-h intervals for three days. No significant difference was seen at 24 h after transfection. However, at both 48 h and 72 h post transfection, cell monolayers transfected with occludin siRNA showed significantly lower TEER (Figure 5A), indicating disruption of barrier integrity in these cells. To examine the distribution of tight junctions, immunofluorescence staining was performed 72 h post transfection. As Figure 5B shows, cells transfected with occludin siRNA showed much lower staining intensity and a more discontinuous distribution compared with siRNA scrambled control. Surprisingly, the membrane localization of claudin-18 was also decreased in occludin knockdown cells (Figure 5B), but claudin-3 and claudin-4 levels were not altered. These in vitro data further support the hypothesis that occludin is required for alveolar barrier function.

To extend these findings that occludin is needed for alveolar barrier function, we asked whether overexpression of occludin could improve barrier integrity by transfecting primary rat type II cells with a plasmid expressing occludin and used TEER measurement to monitor barrier integrity in these cells. Occludin transfection led to a 12.8% increase in TEER (Figure 5C). The intensity and membrane localization of occludin were also increased (Figure 5D). Similar changes were noted for claudin-18, suggesting some form of co-regulation of these two TJ components. 

### 2.5. Gene Transfer of Occludin Can Treat Lungs with Existing LPS-Induced Injury

**Figure 6:**
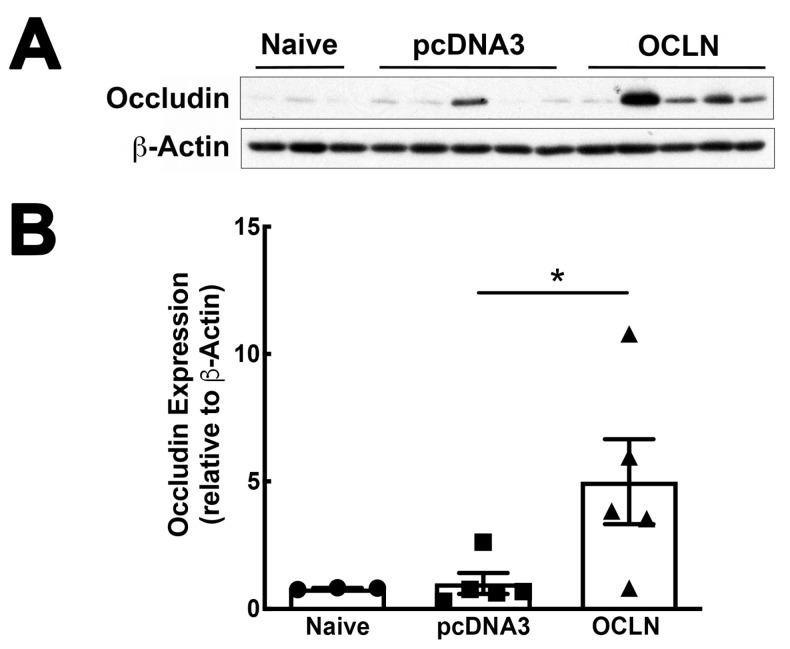
Occludin expression is increased after electroporation-mediated gene delivery. Expression of occludin was determined by Western blot (**A**) in mouse lungs after gene transfer for 2 days (n = 3–5) and quantified by densitometry (**B**). Data are shown as mean ± SEM. Statistics were performed using one-way ANOVA with Tukey’s multiple comparisons test. n = 3–5, * *p* < 0.05.

**Figure 7:**
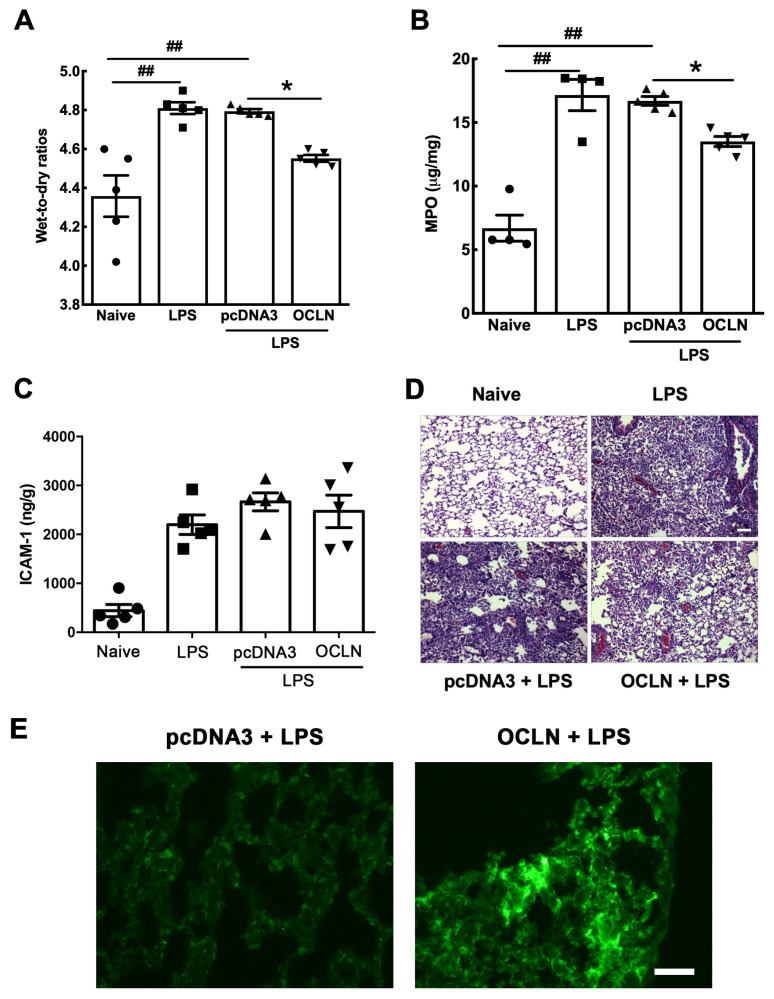
Electroporation-mediated gene transfer of occludin can treat LPS-injured lungs. LPS (5 mg/kg) was intratracheally administered to mice and, 1 day later, 100 μg of plasmid in 50 μL was delivered to the lungs by electroporation at 200 V/cm using 8 pulses of 10 msec in duration. Two days later, lungs were removed for gravimetric analysis or analysis of total lung homogenates. (**A**) Wet-to-dry ratios, (**B**) MPO activity in total lung, and (**C**) ICAM-1 levels in total lung are shown as mean ± SEM (n = 5–6). Statistical analysis was by one way ANOVA followed by post-hoc analysis using Tukey’s test. ^##^ *p* < 0.01 compared to naïve and * *p* < 0.05 compared to pcDNA3. (**D**) Hematoxylin and eoxin staining were used to compare the histologic features. Scale bar: 100 µm. (**E**) Occludin levels (green) were evaluated using immunofluorescence in animals that received either empty plasmids (pcDNA3) or occludin (OCLN)-overexpressing plasmids one after injury by LPS administration. Scale bar: 40 µm.

So far, our data have clearly established an indispensable role of occludin in lung barrier function. We next explored whether occludin could be used as a target to treat pre-existing lung injury. Gene delivery of plasmids encoding the mouse occludin gene lead to a four-fold increase of its expression in the total lung in mice compared with naïve mice while no increase was seen in mice receiving the pcDNA3 control plasmid (Figure 6A,B). These data indicate that delivered transgenes can be expressed efficiently in mouse lungs. Next, we investigated whether restoration of occludin levels could be used to rescue mice with existing lung injury. Mouse lungs were injured by intratracheal administration of LPS (5 mg/kg) and, one day later, the plasmid expressing occludin was electroporated into the lungs. Two days later, injury was assessed by measurement of wet-to-dry ratios, myeloperoxidase (MPO) levels, histology, BAL albumin levels, BAL cellularity, and primary neutrophil infiltration. The wet-to-dry weight ratio was increased from 4.36 ± 0.11 in naïve lungs to 4.81 ± 0.03 in mice injured with LPS. Remarkably, mice receiving occludin gene delivery showed significantly less pulmonary edema compared to those receiving pcDNA3 control (Figure 7A). To determine the inflammatory response induced by LPS, we measured the MPO levels by enzyme-linked immunosorbent assay (ELISA) in mouse lung tissues. As shown in Figure 7B, LPS administration resulted in a 2.5-fold increase in the levels of MPO compared with naïve lungs. Reduced MPO levels were found in mice that received occludin plasmids compared with LPS-challenged mice alone or LPS-challenged mice that received the empty plasmid pcDNA3. Levels of ICAM-1 were also measured by ELISA in total lung homogenates to evaluate the effects of occludin overexpression on endothelial cell inflammation. However, while ICAM-1 increased in LPS treated animals compared to control naïve mice, overexpression of occludin showed no significant reduction in expression (Figure 7C). Histological evaluation revealed mild edema and somewhat reduced airspaces in mice that received a plasmid expressing occludin, whereas there was severe edema, thickened alveolar walls, and high numbers of infiltrating cells in mice that received the control pcDNA3 plasmid or LPS alone (Figure 7D). Finally, immunofluorescence staining confirmed that occludin was indeed overexpressed in the alveolar epithelium following delivery and electroporation of the occludin plasmids compared to animals that received empty plasmid (Figure 7E) [19,20,21]. 

**Figure 8:**
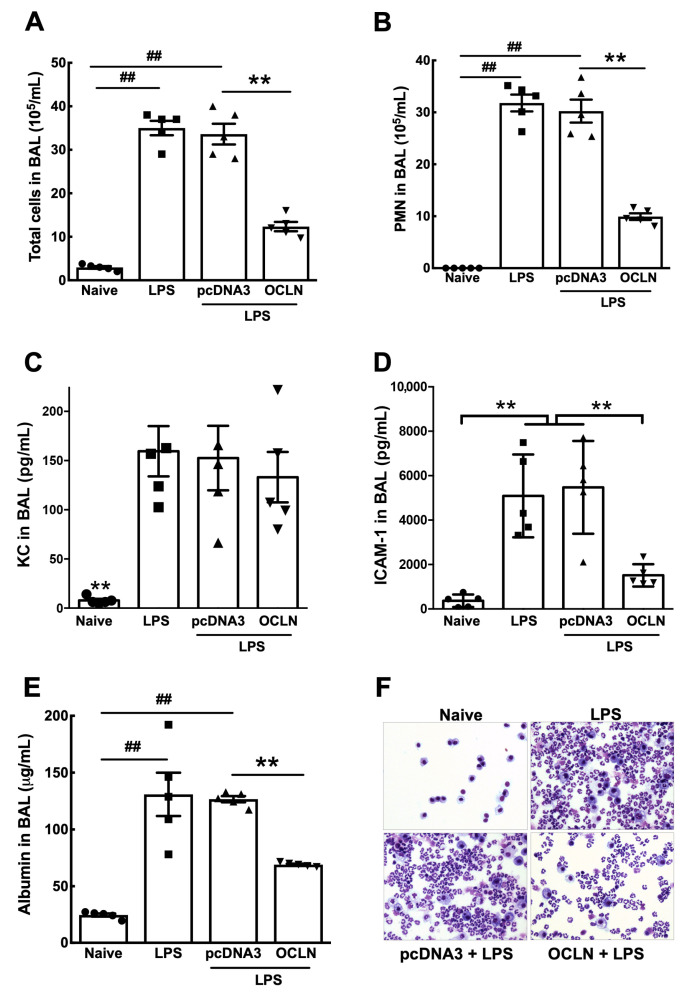
Electroporation-mediated gene transfer of occludin reduces inflammation in BAL fluid. LPS (5 mg/kg) was intratracheally administered to mice and, 1 day later, 100 μg of plasmid in 50 μL was delivered to the lungs by electroporation at 200 V/cm using 8 pulses of 10 ms in duration. Two days later, BAL fluid was collected and assessed by (**A**) cellularity of BAL fluid, (**B**) infiltration of PMNs in BAL, (**C**) expression of KC in BAL, (**D**) levels of soluble ICAM-1 released into BAL, and (**E**) albumin in BAL. Statistical analysis was by one way ANOVA followed by post-hoc analysis using Tukey’s test. ^##^ *p* < 0.01 compared to naïve and ** *p* < 0.01 compared to pcDNA3. (**F**) Representative photographs show PMN infiltration in BAL fluid.

To determine whether this reduced pulmonary edema was accompanied by decreased neutrophilic inflammation, cells present in the bronchoalveolar space were assessed in BAL fluid by cytospin analysis. As shown in Figure 8A,B, total cells and polymorphonuclear cells (PMNs) in BAL fluid were increased ten-fold due to an increase in the number of neutrophils after LPS exposure compared with naïve mice. As can be seen, transfer of pcDNA3 after LPS instillation resulted in no change in cellularity of BAL fluid compared to LPS only mice. By contrast, transfer of occludin plasmids significantly decreased the numbers of total cells and PMNs in BAL fluid. Importantly, occludin gene transfer markedly reduced PMNs to 9.93 ± 0.64 (×10^5^/mL), compared to 30.24 ± 2.21 (×10^5^/mL) of the control pcDNA3 (Figure 8B). However, gene transfer of occludin did not greatly reduce expression of the pro-inflammatory chemokine KC compared to mice receiving only LPS or pcDNA3 after LPS administration (Figure 8C). Occludin overexpression following LPS administration did significantly reduce levels of released ICAM-1 in the BAL fluid (1513 ± 225) compared to that in mice that received either LPS alone or pcDNA3 after LPS (5089 ± 833 and 5472 ± 934, respectively) (Figure 8D). Similarly, gene transfer of occludin also decreased albumin levels in BAL fluid to 69.14 ± 0.79, compared to 126.7 ± 2.74 in mice that received pcDNA3 after LPS administration (Figure 8E). No difference was observed between LPS-injured mice alone or LPS-injured mice that received the control plasmid pcDNA3. Respective cytospin images of these observations are shown in Figure 8F. In sum, these data suggest that occludin is sufficient to increase barrier function and that by increasing its levels in the lung, it can be used to treat acute lung injury.

## 3. Discussion

While important for TJ integrity in some tissues such as the brain, gut, and colon [13,22], occludin appears to be functionally redundant in other tissues [10,11,23]. Whether it plays a dispensable role in the lung epithelium is unknown. Here, we provide the first unequivocal evidence that occludin is needed for lung alveolar barrier function. Occludin is decreased in alveolar epithelial cells in ARDS patients and knockdown of occludin in mice recapitulated phenotypic characteristics of ARDS, including higher lung wet/dry ratio, worse lung histology, and higher total BAL protein. Conversely, overexpression of occludin improves these lung inflammation and injury outcomes in mice with existing lung injury. In addition, TEER and immunofluorescence staining in primary cells found that occludin level is positively correlated with epithelial barrier integrity. Taken together, our data reveal that occludin is an indispensable regulator of alveolar barrier function and show the therapeutic utility of targeting it against acute lung injury.

Although occludin was known to have decreased expression in cells and lungs of animals challenged with LPS [15], hypoxia [6], or bacterial infection [24], direct evidence from ARDS patients is limited. By using immunofluorescence staining of human donors, we demonstrated that occludin is reduced in alveolar epithelial cells in ARDS patients. Similar reduction was observed for claudin-4 and claudin-18. Claudin-4 knockout mice have increased susceptibility to lung injury despite of normal physiological phenotype [25]. Claudin-18 knockout mice exhibit dysregulated alveolar epithelial TJ composition and barrier permeability [26]. Future work should explore if modulating levels of these two TJ proteins will have similar therapeutic effect as occludin for the treatment of ARDS. Similar findings that occludin levels are decreased in the lungs of ARDS patients has also been reported by recently in a study looking at the role of miR-193b-5p. dos Santos et al. found that at least one of the targets of miR-193b-5p, an miRNA whose expression is increased in lungs from ARDS patients, is occludin and that inhibition of miR-193b-5p using oligonucleotides prevents the decreases in occludin levels seen in the lungs of mice following LPS-induced lung injury as well as in cultured human microvascular endothelial cells exposed to TNFα [27]. However, in these studies, the effects of decreased occludin alone in the absence of any injurious stimulation (e.g., TNFα or LPS) was not investigated.

Our data reveal that occludin knockdown increases lung wet-to-dry ratio, but to a lesser extent than LPS treatment only. The difference may be attributed to the broad effect of LPS. Via a TLR-4 dependent pathway, LPS is known to induce the release of a number of cytokines that eventually leads to the damage of many tight junction proteins [28,29]. Therefore, it is not surprising that LPS led to greater damage to the lung epithelium than knockdown of occludin alone. For the same reason, occludin overexpression in LPS-injured mice only partially rescued the lung inflammation and injury (Figure 7 and Figure 8). These results highlight the complexity of ARDS pathogenesis. Targeting multiple genes and pathways may be required to maximize the efficacy of treatment as we have done by gene transfer of either the β1 subunit of the Na^+^,K^+^-ATPase or MRCKα [21,30]. Nevertheless, occludin appears to be a promising target to treat ARDS. Indeed, Zhou et al. have demonstrated that solnatide, a synthetic peptide that has profound therapeutic activity, can increase occludin expression, thereby improving lung barrier function [31]. Another study also shows that upregulation of occludin using protein kinase C inhibitor can reduce ventilation-induced lung injury [9]. Our results substantiate these findings and provide rationale for future research to utilize occludin for ARDS treatment.

Our previous results have demonstrated that electroporation is an effective approach for gene transfer to the lung [19,20,21,32,33]. The current study not only confirms this, but also extends its application for gene knockdown. Therefore, electroporation provides a quick and effective experimental tool to analyze gene function in animal models. One limitation of this study is that the in vivo gene delivery using electroporation did not lead to knockdown or overexpression of occludin in all cells of the alveoli. Quantitation of the number of cells expressing transgene after electroporation in the lung reveals that almost one third of total cells in the lung show productive transfection and expression. If an even greater number of cells were transfected, we would observe an even more significant reduction in occludin expression following delivery of the shRNA plasmids and an even greater difference in lung inflammatory injury. Enhancing the efficiency of gene delivery, extending the duration of transgene expression, or the use of conditional and inducible knockout models may allow further confirmation of our conclusions. In addition, simultaneous knockdown of occludin and overexpression of other claudins, such as claudin-18, will help determine whether the effect of occludin on lung function is mediated directly though occludin itself or indirectly via modulation of other claudins. In either case, however, our data clearly demonstrate the indispensable role of occludin in lung epithelial barrier function. 

A possible interaction between occludin and claudin-18 is observed from our study (Figure 5B,D). This is consistent with earlier findings that occludin is 42% lower at the protein level in claudin-18 knockout versus wild-type lung [26]. This interaction between different claudins/occludin has been reported previously and is required to recruit claudins to the TJ [34]. For example, claudin-1 and claudin-5 improved enrichment of occludin and tricellulin at cell–cell contacts [35]. The interaction may also affect the ability of claudin to interact with the scaffold protein ZO-1 [36]. Hence, the effect of occludin in alveolar barrier function may involve both its direct structural role in TJ, but also its indirect effect on claudin-18. Future work needs to be carried out to establish whether a direct cis-interaction between occludin and claudin-18 exists.

## 4. Methods

### 4.1. Plasmids and siRNA

The plasmid pcDNA3.1 was from Invitrogen/Thermofisher (Carlsbad, CA, USA, catalog #V79020). pCMV-occludin expresses a GFP-tagged mouse occludin from the CMV promoter (Origene, Rockville, MD, USA, catalog # MG226013). pEGFP-C1 expresses GFP from the CMV promoter (Clontech, Mountain View, CA, USA, catalog # 6084-1). Four distinct RFP-expressing occludin shRNA plasmids (catalog # TF501526) and a scrambled shRNA plasmid (catalog # TR30015) were purchased from Origene (Rockville, MD, USA). The shRNA sequences were: GATGAATTCTTCACTTCTACAAATGGAC, TCCACCTATCACTTCAGATCAACAAAGAC, AGGTACTGGTCTCTACGTGGATCAATATT, and CTATGCGGAAAGAGTTGACAGTCCAATGG. These shRNA expressing plasmids were driven by U6 pol III promoter. Plasmids were purified using Qiagen Giga-prep kits (Qiagen, Chatsworth, CA, USA) and suspended in 10 mM Tris-HCl (pH 8.0), 1 mM ethylenediaminetetraacetic acid, and 140 mM NaCl. siRNA control and siRNA against occludin were purchased from Integrated DNA Technologies (Coralville, IA, USA). 

### 4.2. In-Vivo Gene Transfer and Induction of Acute Lung Injury

Male C57BL/6 mice (9–11 weeks) were anesthetized with isoflurane and 100 μg of the plasmid encoding occludin or 160 μg of a combination of 4 distinct occludin shRNA plasmids were delivered in 50 μL of 10 mM Tris-HCl (pH 8.0), 1 mM EDTA, and 140 mM NaCl, to mouse lungs by aspiration. Eight 10 msec square wave pulses at a field strength of 200 V/cm were immediately applied using cutaneous electrophysiology electrodes (Medtronic, Redmond, WA, USA) placed on the mouse chest. Pulses were delivered with an ECM830 electroporator (BTX, Harvard Apparatus, Holliston, MA, USA) [19,20,21,32]. All LPS-challenged mice received 5 mg/kg of LPS (*Escherichia coli* O55:B5, 15,000,000 endotoxin units/mg protein; Sigma-Aldrich, St. Louis, MO, USA) in 50 μL of phosphate-buffered saline (PBS) by aspiration, one day before gene transfer (overexpression of occludin) or 4 days after gene delivery (knockdown of occludin, n = 5 mice/group). All experimental procedures were performed in accordance with institutional guidelines for the care and use of laboratory animals in an American Association for the Accreditation of Laboratory Animal Care-approved facility.

### 4.3. Quantitation of In Vivo Gene Transfer and Expression Efficiency

pEGFP-C1 plasmid was transferred to mouse lungs (n = 6) by electroporation as described. Two days later, lungs were perfused and inflated with 20 cc/kg aqueous buffered formalin immediately following euthanasia and used for paraffin-embedding. Paraffin-embedded tissue sections (5 µm) were cut from the upper, middle, and lower portions of the lungs of each animal and were rehydrated and subjected to antigen retrieval in Tris-HCl buffer (100 mM, pH 9.5). Sections were immunostained overnight with FITC conjugated anti-GFP antibody (Abcam, Cambridge, MA, USA, catalog #AB6662) as previously described [37]. Cells expressing GFP were counted in a total of 10 sections from each animal (representing the three portions of the lung) and used for quantification of expression.

### 4.4. Isolation, Culturing and Transfection of Rat Alveolar Type II Cells

Isolation of alveolar type II cells was performed using an IgG-panning approach as previously described [38]. Briefly, lungs from Sprague Dawley rats (200–250 g) were surgically removed, perfused, and lavaged. Epithelial cells were then released from the lung using 1 mg/ml of elastase (Worthington Biochemical, Lakewood, NJ, USA). After IgG panning step to remove the white blood cells, the cells were resuspended in DMEM containing 10% FBS and plated on fibronectin coated plates. ATII cells were cultured for 48 h to allow them to differentiate into ATI cells. Electroporation-mediated transfection of ATI cells was carried out using the same protocols as previously described [21]. The optimal conditions were one square wave pulse at 300 V, 2000 μF, 1000 Ω, and 20 ms.

### 4.5. TEER Measurement

Prior to the assay, cells were cultured on 12-well transwell plates (12 mm transwell with 0.4 μm pore polyester membrane insert; Corning, Corning, NY, USA) and then were moved to the tissue culture hood for 15 min to allow the medium to equilibrate to room temperature. TEER was measured using an epithelial voltmeter (EVOM2; World Precision Instruments, Sarasota, FL, USA). Three readings were recoded and averaged for each well. To calculate TEER, the resistance of the fibronectin-coated insert without cells (blank resistance) was subtracted from the measured resistance, then multiplied by the surface area of the insert (1.12 cm^2^ for the 12-well plates used).

### 4.6. Western Blot Analysis

Western blots were performed as previously described [39]. Briefly, lung tissues were solubilized in lysis buffer containing protease inhibitor. Thirty μg of total protein was loaded on 10% SDS-PAGE, transferred to PVDF membrane, and probed with primary antibodies against occludin (1:125; catalog #71-1500, Thermo Fisher Scientific, Waltham, MA, USA) or β-actin (Sigma-Aldrich, St. Louis, MO, USA). Data were analyzed using NIH Image J software, version 1.53t. 

### 4.7. Measurement of Wet-to-Dry Ratios

Wet-to-dry ratios were determined as previously described [21]. The effect of LPS-induced acute lung injury on total lung water content was determined at 72 h after instillation of LPS. Mice were exsanguinated via laceration of left renal artery and vein, and then lungs were excised, and surface liquid was blotted away. Wet lung weight was assessed, and a stable dry weight was obtained after lungs were placed in a hybridization oven at 70 °C for 72 h.

### 4.8. Bronchoalveolar Lavage (BAL) Analysis

BAL was performed as described previously [20]. Briefly, two separate 0.7 mL aliquots of sterile PBS were instilled into mouse lungs for lavaging. The fluid was placed on ice for immediate processing and the total number of cells in the lavage was determined using a hemocytometer. Albumin in mouse BAL was measured by ELISA according to the manufacturer’s instruction (ALPCO, Salem, NH, USA, catalog # 41-ALBMS-E01) and cells from BAL were stained with Diff-Quik™ (Siemens, Newark, DE, USA, catalog # 10736133) after cytospin.

### 4.9. Histological Analysis

Lungs were inflated with 20 cc/kg 10% (*v*/*v*) buffered zinc formalin (Z-FIX; Anatech, Battle Creek, MI, USA) immediately following euthanasia and used for paraffin-embedding. Sections (5 µm) were stained with hematoxylin and eosin, blinded, and reviewed for analysis of inflammatory response and pathological changes in the lung according to our previous studies [21]. 

### 4.10. Immunofluorescence Staining

Human tissues were obtained from the Department of Pathology at the University of Rochester and studies were approved by the University of Rochester Institutional Review Board and Office for Human Subjects Protection. Immunofluorescence staining was performed as described previously [37]. Human and mouse paraffin-embedded tissue sections (5 µm) were rehydrated and subjected to antigen retrieval in Tris-HCl buffer (100 mM, pH 9.5) by heating them with steam for 30 min in a steamer. Slides were then cooled for 30 min to room temperature, washed for 1 min at room temperature with Tris-HCl buffer (pH 9.5), followed by a 5 min wash in distilled water and incubation with Proteinase K for 15 min. Sections were washed successively for 5 min each in water, PBS, and PBS containing 0.1% Triton X-100, prior to blocking with DAKO serum-free protein block reagent (Agilent, Santa Clara, CA, USA) for 1 h at room temperature in a humidified chamber, according to the manufacturer’s recommendations. Sections were washed three times for five minutes each with PBS containing 0.1% Triton X-100 and sections were immunostained overnight with rabbit anti-occludin antibody (1:50; catalog #71-1500, Thermo Fisher Scientific, Waltham, MA, USA), rabbit anti-claudin-4 (Invitrogen, Camarillo, CA, USA, catalog #36-4800), and rabbit anti-claudin-18 (Invitrogen, Camarillo, CA, USA, catalog #38-8000). The immune complexes were detected using Alexa Fluor 555 conjugated goat anti-rabbit secondary antibody (1:500; catalog #A-21428, Invitrogen, Grand Island, NY, USA) before sections were counterstained with DAPI. Stained sections were visualized using a Leica DM RXA2 microscope (Leica, Wetzlar, Germany) using 5×, 10×, 20×, or 40× Leica objectives and photographed with a SPOT color CCD camera (SPOT Imaging, Sterling Heights, MI, USA).

### 4.11. Statistical Analysis

Quantitative results are expressed as mean ± SEM for in vivo studies and mean ± SD for in vitro studies. When greater than two conditions were analyzed, the data were evaluated statistically with one-way ANOVA and significance between groups was determined post-hoc using Tukey’s test (Prism 10 version 10.2.0 GraphPad Software, San Diego, CA, USA). For comparison of two conditions only, a student’s *t*-test was used. *p*-values < 0.05 were considered statistically significant.

## Data Availability

The original contributions presented in the study are included in the article/Supplementary Materials, further inquiries can be directed to the corresponding author.

## Supplementary Materials

The following supporting information can be downloaded at: https://www.mdpi.com/article/10.3390/ijms252111595/s1.
