# Supplementary material for: Occludin Is Essential to Maintain Normal Alveolar Barrier Integrity and Its Protective Role During ARDS Progression
Source: Int J Mol Sci. 2024 Oct 29;25(21):11595. doi: 10.3390/ijms252111595 (PMC11546927; doi:10.3390/ijms252111595)
Supplement: Supplementary file 1 [file ijms-25-11595-s001.zip › ijms-3278019-supplementary.pdf]

Figure S1

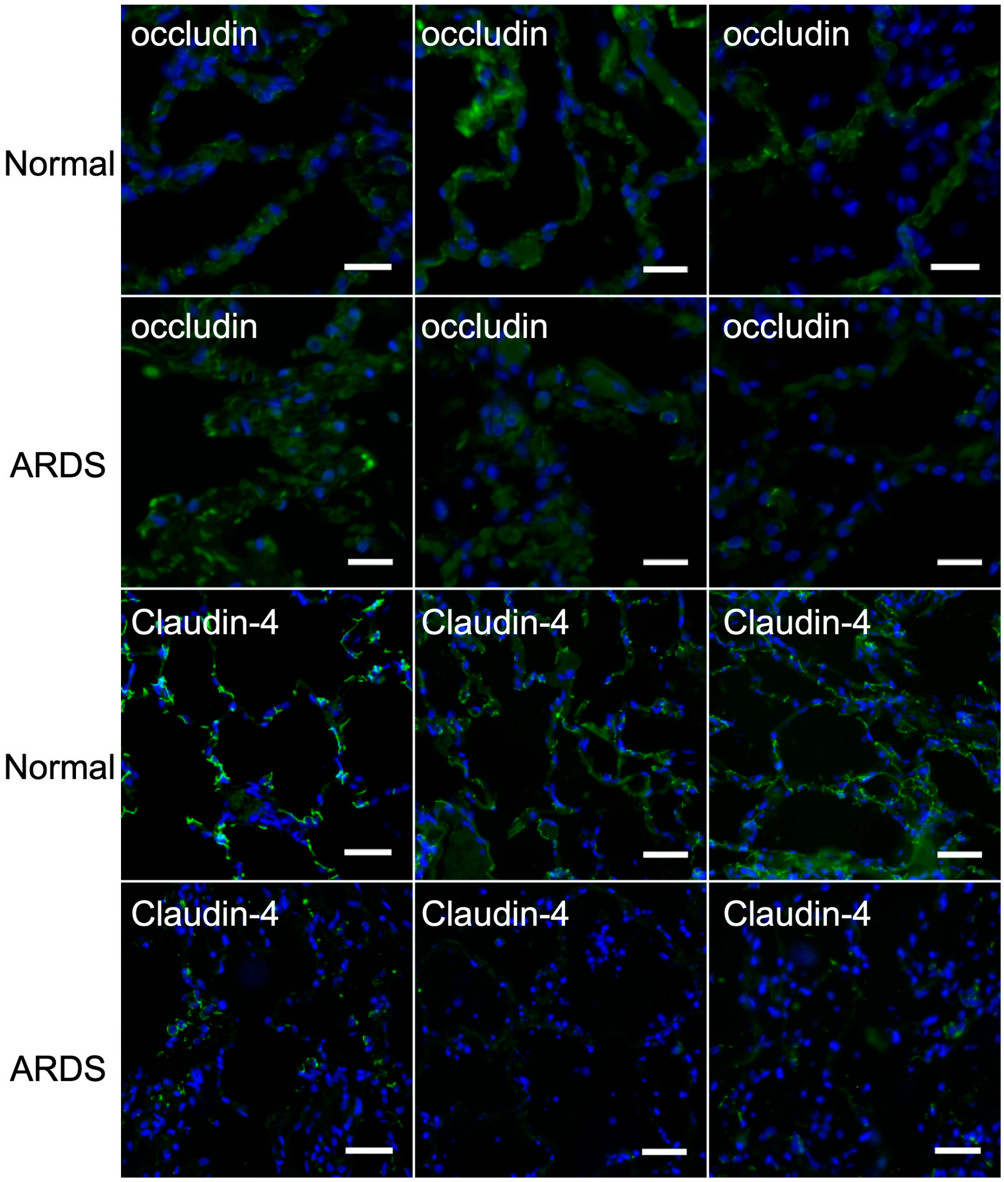

Figure S1. Changes of tight junction expressions in ARDS. The expression of occludin and claudin-4 f are decreased in the lungs from 3 different patients with ARDS compared with 3 healthy controls. Representative images show nuclei (blue) and reduced occludin and claudin-4 expression (green). Scale bar: 40 um.

.

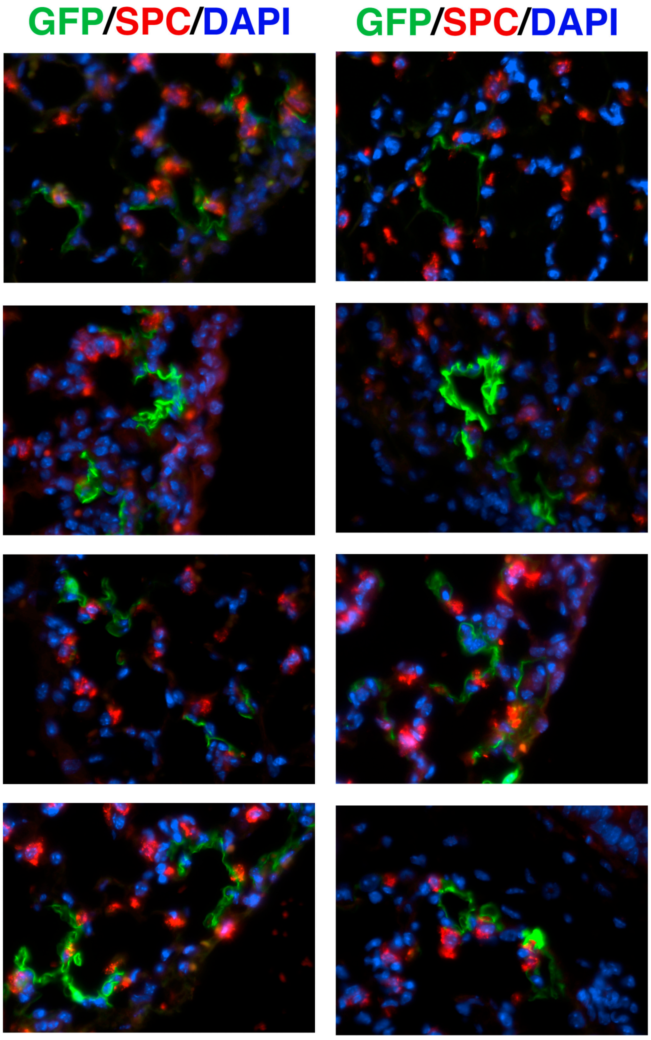

Figure S2. Efficiency of electroporation-mediated lung gene transfer. The lungs of mice (n=6) received 100 µg of pEGFP-C1 plasmid by electroporation. Expression was quantified in paraffin sections of inflation-fixed lungs 2 days later. The number of GFP-expressing cells (green) in 10 sections per mouse lung (representing upper, middle, and lower portions of each lung) were counted and used to determine the efficiency of transfer and expression (compared to total number of cells, as determined by DAPI staining, blue). Alveolar epithelial type II cells were stained with antibodies against SPC (red). Representative images from multiple animals are shown. An average of 33.2 ± 3.5% of cells (18 to 52% range) expressed GFP.
